# Deviation From Optimal Physical Activity Duration Worsens Depressive Symptoms Through High Sleep Reactivity and Poor Sleep Quality in Adult Volunteers From the Community

**DOI:** 10.1002/ejsc.70111

**Published:** 2025-12-17

**Authors:** Tomoteru Seki, Akiyoshi Shimura, Kazuki Nakajima, Masayuki Kikkawa, Chihiro Morishita, Yu Tamada, Mina Honyashiki, Jiro Masuya, Takeshi Inoue

**Affiliations:** ^1^ Department of Psychiatry Tokyo Medical University Shinjuku‐ku Japan; ^2^ Department of Psychiatry Tokyo Medical University Hachioji Medical Center Hachioji‐shi Japan

**Keywords:** depressive symptoms, path model, physical activity, sleep quality, sleep reactivity

## Abstract

Physical inactivity is considered to increase depressive symptoms. There are also studies reporting that overly strenuous physical activity can negatively affect a person's mental state. Insomnia, which is associated with lower levels of physical activity and increased sleep reactivity, is also known to be associated with depression. However, the directionalities of the associations among these factors remain unclear. The aim of this study was to investigate whether both insufficient and excessive physical activity affect depressive symptoms through sleep reactivity and sleep quality. Between April 2017 and April 2018, self‐administered surveys were provided to adult volunteers through our acquaintances at Tokyo Medical University. The study included 526 volunteers whose responses were considered valid. Demographic information, results of the International Physical Activity Questionnaire, the Ford Insomnia Response to Stress Test (FIRST), the Pittsburgh Sleep Quality Index, and the Patient Health Questionnaire‐9 (PHQ‐9) were used for analysis. Quadratic regression was performed to examine whether the PHQ‐9 score was significantly explained by physical activity duration in a U‐shaped curve. Path analysis was performed to identify indirect effects. Quadratic regression indicated the presence of an optimal physical activity duration (25.7 h/week) that was related to the lowest PHQ‐9 score, and both insufficient and excessive physical activity could be expressed as a Difference from Optimal Physical Activity Duration (DOP). Path analysis demonstrated that DOP increased the PHQ‐9 score through the FIRST score.These findings indicate that deviation from the optimal physical activity duration worsens depressive symptoms through high sleep reactivity.

## Highlights

- Both insufficient and excessive physical activity duration increased depressive symptoms, forming a U‐shaped curve relationship. In adult volunteers from the community, the optimal physical activity duration to minimize depressive symptoms was 25.7 h per week.
- Sleep quality, sleep reactivity, subjective social status, and past psychiatric history independently influence depressive symptoms, as demonstrated by multiple linear regression.
- Path analysis revealed that deviation from the optimal physical activity duration worsens depressive symptoms through increased sleep reactivity. This highlights the importance of performing physical activity at an optimal duration and targeting sleep reactivity to improve depressive symptoms.

## 1 Introduction

Physical activity is generally recognized as having a positive impact on mental state, and its association with depression, which is one of the most common and serious mental health conditions (Remes et al. 2021), is more strongly supported by previous research (Kaseva et al. 2019) than its association with other mental health indicators, such as anxiety. For example, various studies have demonstrated the association between higher physical activity and lower depression (De Moor et al. 2006; Paffenbarger Jr et al. 1994; Panagiotakos et al. 2008). Therapeutic interventions involving exercise have been shown to reduce the severity of depression in patients (Josefsson et al. 2014; Schuch et al. 2016) and prevent depressive symptoms, even among the general population (Brown et al. 2005; Chekroud et al. 2018; de Zeeuw et al. 2010; Teychenne et al. 2008). However, it remains unclear as to what level of physical activity most efficiently improves depressive symptoms. Physical inactivity is known to contribute to depressive symptoms (Cheval et al. 2022). On the other hand, some studies have reported that excessive physical activity can negatively affect mental health (Johnson and Thiese 1992). These seemingly contradicting results suggest that there is an optimal physical activity level that will maximize mental health improvements. In fact, previous studies have suggested that there may be a U‐shaped or L‐shaped association between the level of physical activity and mental state (Chekroud et al. 2018; Y. S. Kim et al. 2012).

In addition to physical activity, improving sleep quality also leads to a substantial positive effect on the mental state. For example, a meta‐analysis of randomized controlled trials (RCTs) identified a dose‐dependent association in which greater enhancements in sleep quality were associated with greater improvements in mental health (Scott et al. 2021). On the other hand, insomnia, which is marked by a difficulty in the onset or maintenance of sleep, resulting in impairment of daytime functioning, is known to be strongly associated with an increased risk for developing depression (Baglioni et al. 2011; Cheval et al. 2022; Ford and Kamerow 1989; Jaussent et al. 2011; Ohayon 2002; Okajima et al. 2012; Pigeon et al. 2008). In addition, insomnia has been reported to be strongly associated with mental health disorders and is also a risk factor for developing suicidal ideations and behavior (Baglioni et al. 2011; Bjørngaard et al. 2011; Pigeon et al. 2012).

Among the many factors that increase an individual's vulnerability to insomnia (C. L. Drake and Roth 2006), exposure to stressors is well‐known to promote the onset of insomnia. Stress‐diathesis models of insomnia, such as the Spielman 3P model (Spielman et al. 1987), are extensively referenced in the literature. The association between exposure to stressors and subjective and objective sleep quality has been demonstrated across various psychosocial stressors (Akerstedt 2006; Hall et al. 2008, 2007; Kalimo et al. 2000; E. J. Kim and Dimsdale 2007; Taylor et al. 2016; Verlander et al. 1999). A study using the chronic caregiving stress model showed that the association between chronic stress and depressive symptoms was mediated by poor sleep quality (da Estrela et al. 2021). Other cross‐sectional, longitudinal, and experimental studies have indicated that an exposure to stressors acts as a risk factor for the onset of sleep disturbances (C. L. Drake et al. 2003; Pillai et al. 2014). Interestingly, there are substantial differences among individuals in the risk of insomnia following exposure to stressors. This suggests that there is a trait‐like vulnerability to stress‐associated insomnia. Such an individual factor is termed sleep reactivity and is gaining increasing attention (C. Drake et al. 2004; C. L. Drake et al. 2011; Jarrin et al. 2013; S. Nakajima et al. 2014). Recently, a study demonstrated a significant association between greater sleep reactivity and more severe depressive symptoms, partially mediated by insomnia (Vargas et al. 2015). Another study reported that premorbid sleep reactivity constitutes a significant risk factor for the onset of insomnia, and furthermore, represents a notable risk factor for the development of depression 2 years subsequently, mediated by insomnia (C. L. Drake et al. 2014). Considering the above, to improve depressive symptoms, it is important to improve not only sleep quality itself but to also target sleep reactivity.

Some studies have demonstrated that physical activity can effectively improve sleep quality. For example, previous studies indicated that physical activity may aid in reducing college students' sleep quality problems (Li and Guo 2023; Wunsch et al. 2017). Other RCTs have validated that exercise positively impacts sleep quality, sleep onset latency, total sleep time, sleep efficiency, and severity of insomnia (Hartescu et al. 2015; Passos et al. 2010; Reid et al. 2010). In contrast, another study clarified an association between lower levels of physical activity and a higher prevalence of insomnia (Morgan 2003). Although many studies have been performed regarding physical activity and sleep quality, as described above, little is known about the relationship between physical activity and sleep reactivity. It can be assumed that physical activity also affects sleep reactivity and that both sleep reactivity and sleep quality play roles in the pathways from physical activity to depressive symptoms. However, the directionalities of the associations among these factors remains unclear.

The aim of this study was to investigate whether both insufficient and excessive physical activity affect depressive symptoms through sleep reactivity and sleep quality. To address this question, we (i) examined whether there is a U‐shaped association between physical activity duration and depressive symptoms, (ii) conducted multiple linear regression (MLR) to control for potential confounding variables associated with depressive symptoms, and (iii) conducted path analyses to test whether deviation from the physical activity duration associated with the lowest depressive symptoms affects depressive symptoms through sleep reactivity and sleep quality. In these analyses, the Patient Health Questionnaire‐9 (PHQ‐9) score was used as the variable representing depressive symptoms. Based on our previous study (Ono et al. 2017), the following variables were selected as potential predictors of depressive symptoms: participant characteristics (age, sex, marital status, education duration, employment status, current physical disease, subjective social status, past psychiatric history, current psychiatric disease, and first‐degree relatives with psychiatric disease), as well as scores from the International Physical Activity Questionnaire (IPAQ), the Ford Insomnia Response to Stress Test (FIRST), and the Pittsburgh Sleep Quality Index (PSQI). We used data from adult volunteers collected using a self‐administered survey between April 2017 and April 2018 through affiliated hospitals of our institution.

## 2 Methods

### 2.1 Subjects

A total of 1237 adult volunteers who were recruited through convenience sampling, targeting workers at facilities associated with Tokyo Medical University and their relatives, were asked to complete self‐administered surveys. The volunteers were assured that their participation in the study was entirely optional and that declining to participate would not result in any disadvantages. They were also notified that the information they provide would be anonymized to ensure that individuals would not be identified. Of all the volunteers, 526 people who provided written consent to participate and valid responses with no missing values were selected as subjects for this study. In this study, demographic information and 4 types of questionnaires as described below were used for analysis. This study was approved by the Ethics Committee of Tokyo Medical University (study approval no.: SH3502), in compliance with the Declaration of Helsinki (amended in Fortaleza in 2013).

### 2.2 Demographic Information

Participant characteristics (age, sex, marital status, education duration, employment status, current physical disease, subjective social status, past psychiatric history, current psychiatric disease, and first‐degree relatives with psychiatric disease) were collected.

### 2.3 Questionnaires

#### 2.3.1 IPAQ

The IPAQ is a self‐reported instrument designed to monitor physical activity among adults (Craig et al. 2003). The short form of the IPAQ consists of seven question items that record physical activity in the following 4 intensity levels: vigorous‐intensity activity, moderate‐intensity activity, walking, and sitting. The total physical activity duration was analyzed. The Japanese short form of the IPAQ, which has been validated (Murase et al. 2002), was used in this study.

#### 2.3.2 FIRST

The FIRST is a self‐reported tool designed to measure sleep reactivity (C. Drake et al. 2004). FIRST consists of 9 question items, each evaluated on a 4‐point scale (1–4), and the total score (9–36) was used for analysis. The Japanese version of the FIRST, which has been validated (S. Nakajima et al. 2014), was used in this study.

#### 2.3.3 PSQI

The PSQI is a self‐rated questionnaire designed to assess subjective sleep quality and disturbances in the previous 1 month (Buysse et al. 1989). The PSQI consists of 18 question items, which are categorized into 7 components. Each component is evaluated on a 4‐point scale (0–3), and the total score (0–21) was used for analysis. The Japanese version of the PSQI, which has been validated (Doi et al. 2000), was used in this study.

#### 2.3.4 PHQ‐9

The PHQ‐9 is a self‐administered instrument designed to evaluate the degree of depression severity (Spitzer et al. 1999). In the present study, the PHQ‐9 was selected because its validity for evaluating depressive symptoms has been established, not only in clinical populations but also in the general population (Martin et al. 2006). It comprises 9 question items, each evaluated on a 4‐point scale (0–3), and the total score (0–27) was used in this study. The Japanese version of the PHQ‐9, which has been validated (Muramatsu et al. 2007), was used in this study.

### 2.4 Statistical Analysis

Simple linear regression and quadratic regression were performed to analyze whether each regression significantly explained the association between physical activity duration and PHQ‐9 score. Pearson correlation coefficients were calculated to estimate the associations between participant characteristics (age, education duration, and subjective social status) or the results of each questionnaire and PHQ‐9 score. The *t*‐test was performed to compare the mean PHQ‐9 scores of the 2 groups regarding participant characteristics (sex, marital status, employment status, current physical disease, past psychiatric history, current psychiatric disease, and first‐degree relative with psychiatric disease). MLR was performed to eliminate potential confounding variables that could be linked to PHQ‐9. Standardized partial regression coefficients (beta) were calculated from standardized variables and represent the strength and the direction of the associations between the variables. The above analyses were conducted using SPSS 28 software (IBM, Armonk, NY, USA).

Path analysis was performed to identify indirect effects. The standardized coefficients (*β*) were calculated from standardized variables and represent the strength and the direction of the associations between the variables. Based on the presumed chronological sequence of the associations, a path model with direct and indirect effects was constructed. Mplus version 8.5 software (Muthén & Muthén, Los Angeles, CA, USA) was used for the analysis.

Differences were considered to be statistically significant when the *p*‐value was less than 0.05 between the 2 groups.

## 3 Results

### 3.1 Simple Linear Regression and Quadratic Regression

**FIGURE 1:**
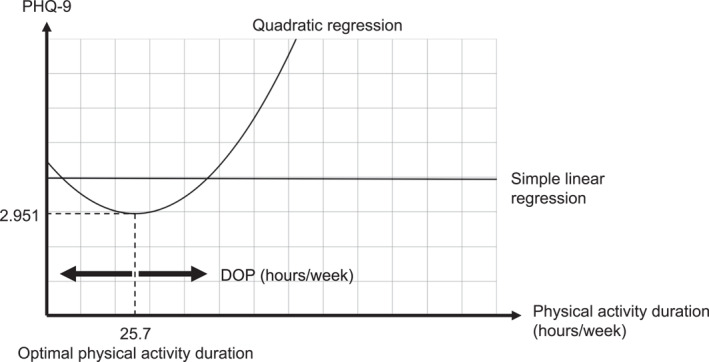
Simple linear regression and quadratic regression. Results of simple linear regression and quadratic regression. DOP, Difference from Optimal Physical Activity Duration; PHQ‐9, Patient Health Questionnaire‐9.

The result of simple linear regression with PHQ‐9 as the dependent variable and physical activity duration as the independent variable was insignificant (PHQ‐9 = −0.000244 physical activity duration + 3.974; *F* = 0.000; *p* = 0.985); therefore, PHQ‐9 was not explained well by the physical activity duration in this model (Figure 1). On the other hand, quadratic regression showed a significant result [PHQ‐9 = 0.002334 physical activity duration^2^—0.119965 physical activity duration + 4.492 = 0.002334 (physical activity duration—25.7)^2^ + 2.951; *F* = 6.906; *p* < 0.001]. Considering the quadratic regression, optimal physical activity duration, that is, the physical activity duration with the lowest PHQ‐9 was 25.7 h/week. Therefore, we introduced a new variable termed the Difference from Optimal Physical Activity Duration (DOP) to evaluate how much an individual's physical activity duration deviated from this optimal duration.

### 3.2 Pearson Correlation Coefficients and the *t*‐Test

The correlations between participant characteristics (age, education duration, and subjective social status) or each questionnaire (DOP, FIRST, and PSQI) score and PHQ‐9 score are shown in Table 1. FIRST and PSQI scores showed moderate to strong positive correlations with the PHQ‐9 score. DOP had a weak positive correlation with the PHQ‐9 score. Education duration and subjective social status demonstrated weak to moderate negative correlations with the PHQ‐9 score. No significant correlation was observed between age and the PHQ‐9 score.

**TABLE 1:** Mean ± SD of characteristics or measures, and their correlation with PHQ‐9 score.

Characteristic or measure	Mean ± SD	Correlation with PHQ‐9 score (*r*)
Age (years)	41.2 ± 11.9	*r* = −0.025, *p* = 0.566
Education duration (years)	14.7 ± 1.8	*r* = −0.089, *p* = 0.040*
Subjective social status (score)	5.1 ± 1.7	*r* = −0.259, *p* < 0.001***
DOP (hours/week)	19.6 ± 7.4	*r* = 0.124, *p* = 0.004**
FIRST (score)	19.1 ± 5.9	*r* = 0.433, *p* < 0.001***
PSQI (score)	5.7 ± 3.5	*r* = 0.553, *p* < 0.001***
PHQ‐9 (score)	4.0 ± 4.2	

*Note:* Data are presented as means ± SD. *r*, Pearson correlation coefficient.

Abbreviations: DOP, Difference from Optimal Physical Activity Duration; FIRST, Ford Insomnia Response to Stress Test; PSQI, Pittsburgh Sleep Quality Index; PHQ‐9, Patient Health Questionnaire‐9; SD, standard deviation.

^*^ *p* < 0.05.

^**^ *p* < 0.01.

^***^ *p* < 0.001.

Comparisons of the PHQ‐9 score between the 2 groups are shown in Table 2. The differences in the PHQ‐9 score of the 2 groups regarding sex, marital status, past psychiatric history, and current psychiatric disease were significant. There was no significant difference in the PHQ‐9 score of the 2 groups regarding employment status, current physical disease, and first‐degree relative with psychiatric disease.

**TABLE 2:** Number of participants for each characteristic, and comparison of PHQ‐9 scores between the 2 groups for each characteristic.

Characteristic	Number	Comparison of PHQ‐9 scores (mean ± SD)
Sex (men:women)	228:298	Men (3.3 ± 3.8) versus women (4.5 ± 4.4), *p* < 0.001***
Marital status (married:unmarried)	346:176	Married (3.5 ± 3.9) versus unmarried (5.0 ± 4.4), *p* < 0.001***
Employment status (employed:unemployed)	514:9	Employed (4.0 ± 4.2) versus unemployed (3.3 ± 5.1), *p* = 0.643
Current physical disease (yes:no)	103:423	Yes (4.2 ± 4.7) versus no (3.9 ± 4.0), *p* = 0.461
Past psychiatric history (yes:no)	62:464	Yes (6.7 ± 5.4) versus no (3.6 ± 3.8), *p* < 0.001***
Current psychiatric disease (yes:no)	21:496	Yes (8.1 ± 5.0) versus no (3.8 ± 4.1), *p* < 0.001***
First‐degree relative with psychiatric disease (yes:no)	53:422	Yes (4.5 ± 4.2) versus no (3.9 ± 4.2), *p* = 0.326

*Note:* Data are presented as means ± SD.

Abbreviations: PHQ‐9, patient health questionnaire‐9; SD, standard deviation.

^***^ *p* < 0.001.

### 3.3 MLR

Table 3 displays the results of MLR, with the PHQ‐9 score as the dependent variable. Using the forced entry method, the following 4 out of the 13 independent variables were found to be statistically significant: PSQI score (*β* = 0.395; *p* < 0.001), FIRST score (*β* = 0.225; *p* < 0.001), subjective social status (beta = −0.152; *p* < 0.001), and past psychiatric history (*β* = 0.087; *p* = 0.048). The remaining 9 independent variables, including DOP, did not have significant effects on depressive symptoms. The adjusted version of the square of the multiple correlation coefficient (*R* ^2^) value was 0.406. The variance inflation factors ranged from 1.042 to 1.650, indicating the absence of multicollinearity.

**TABLE 3:** Results of multiple linear regression analysis with PHQ‐9 score as the dependent variable.

Independent variable	Standardized partial regression coefficient	*p*‐value	VIF
PSQI (score)	0.395	< 0.001***	1.369
FIRST (score)	0.225	< 0.001***	1.329
Subjective social status (score)	−0.152	< 0.001***	1.296
Past psychiatric history (yes:no)	0.087	0.048*	1.417
DOP (hours/week)	0.066	0.084	1.077
Marital status (married:unmarried)	−0.066	0.109	1.247
Employment status (employed:unemployed)	0.043	0.253	1.049
Age (years)	−0.035	0.466	1.650
Sex (men:women)	0.024	0.557	1.256
Current psychiatric disease (yes:no)	0.015	0.727	1.371
Education duration (years)	0.015	0.732	1.488
Current physical disease (yes:no)	0.005	0.904	1.256
First‐degree relative with psychiatric disease (yes:no)	0.001	0.984	1.042
Adjusted *R* ^2^ = 0.406		*F* = 23.946, *p* < 0.001***	

*Note: R* ^2^, square of multiple correlation coefficient; *F*, ratio of mean square regression divided by mean square error.

Abbreviations: DOP, Difference from Optimal Physical Activity Duration; FIRST, Ford Insomnia Response to Stress Test; PSQI, Pittsburgh Sleep Quality Index; PHQ‐9, Patient Health Questionnaire‐9; VIF, variance inflation factor.

^*^ *p* < 0.05.

^***^ *p* < 0.001.

### 3.4 Path Analysis

**FIGURE 2:**
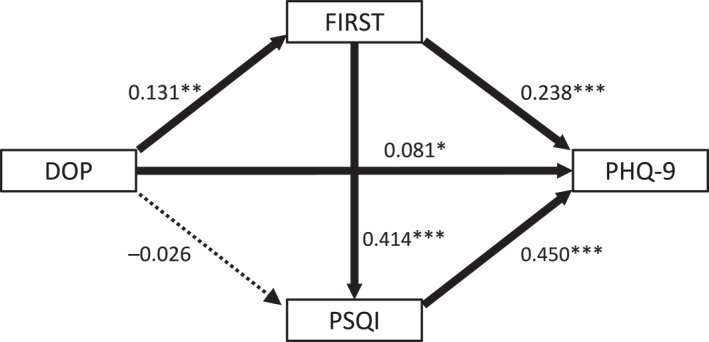
Direct effects in the path model among DOP, FIRST score, PSQI score, and PHQ‐9 score. Results of direct effects in the path model among DOP, FIRST score, PSQI score, and PHQ‐9 score. Rectangles represent observed variables. The arrows with solid lines indicate statistically significant pathways, and the arrow with a dotted line indicates the insignificant pathway. The numbers next to the arrows are the direct standardized coefficients. *p < 0.05, **p < 0.01, ***p < 0.001. DOP, Difference from Optimal Physical Activity Duration; FIRST, Ford Insomnia Response to Stress Test; PSQI, Pittsburgh Sleep Quality Index; PHQ‐9, Patient Health Questionnaire‐9.

**FIGURE 3:**
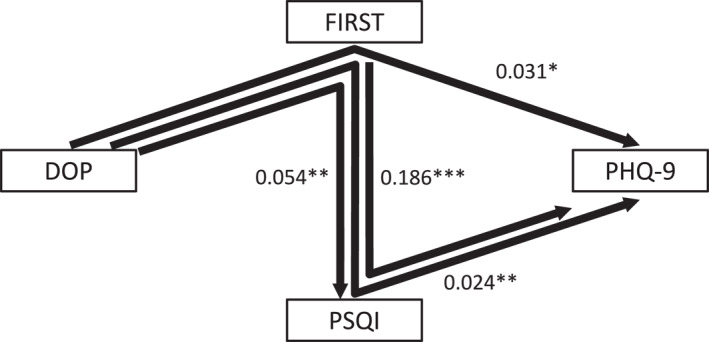
Indirect effects in the path model among DOP, FIRST score, PSQI score, and PHQ‐9 score. Results of indirect effects in the path model among DOP, FIRST score, PSQI score, and PHQ‐9 score. Rectangles represent observed variables. The arrows with solid lines indicate statistically significant pathways, and insignificant pathways are not shown. The numbers next to the arrows are the indirect standardized coefficients. *p < 0.05, **p < 0.01, ***p < 0.001. DOP, Difference from Optimal Physical Activity Duration; FIRST, Ford Insomnia Response to Stress Test; PSQI, Pittsburgh Sleep Quality Index; PHQ‐9, Patient Health Questionnaire‐9.

In the models shown in Figures 2 and 3, the indices suggested a good fit (the comparative fit index = 1.000, and the root‐mean‐square error of approximation = 0.000). The *R* ^2^ value of PHQ‐9 was 0.361, indicating that this model accounts for 36.1% of the variability in the PHQ‐9 score.

#### 3.4.1 Direct Effects

Five direct effects were significant (Figure 2). DOP showed significant positive direct effects on FIRST (*β* = 0.131; *p* = 0.005) and PHQ‐9 scores (*β* = 0.081; *p* = 0.030). FIRST score had significant positive effects on PSQI (*β* = 0.414; *p* < 0.001) and PHQ‐9 scores (*β* = 0.238; *p* < 0.001), and the PSQI score showed a significant positive effect on the PHQ‐9 score (*β* = 0.450; *p* < 0.001).

#### 3.4.2 Indirect Effects

Four significant indirect effects were identified (Figure 3). First, DOP showed a significant positive indirect effect on the PHQ‐9 score through the FIRST score (*β* = 0.031; *p* = 0.012). In addition, DOP also had a significant positive indirect effect on the PHQ‐9 score through a combined pathway involving both FIRST and PSQI scores (*β* = 0.024; *p* = 0.008). In contrast, the indirect effect of DOP on PHQ‐9 scores through the PSQI score alone was not significant (*β* = −0.012; *p* = 0.512, not shown in Figure 3). Thus, the estimated total indirect effects of DOP on the PHQ‐9 score was 0.044, and the estimated total of both direct and indirect effects was 0.124. This indicates that the indirect effect through the FIRST score only contributed to 25.0% of the total effect from DOP on the PHQ‐9 score, and the indirect effect through both FIRST and PSQI scores accounted for 19.4%. These proportions of the indirect effects relative to the total effect remained unchanged even after including subjective social status and past psychiatric history, which were identified to be significant in MLR, as covariates. Furthermore, DOP showed a significant positive indirect effect on the PSQI score through the FIRST score (*β* = 0.054; *p* = 0.007), and the FIRST score showed a significant positive indirect effect on the PHQ‐9 score through the PSQI score (*β* = 0.186; *p* < 0.001).

## 4 Discussion

The directionalities of the associations among physical activity, sleep reactivity, insomnia, and depression remain unclear. The aim of this study was to investigate whether both insufficient and excessive physical activity affect depressive symptoms through sleep reactivity and sleep quality.

### 4.1 Optimal Physical Activity Duration Minimizes Depressive Symptoms

One of the main findings of this study is that the quadratic regression indicated 25.7 h/week as the optimal physical activity duration that minimizes depressive symptoms in adult volunteers from the community and that excessive physical activity and physical inactivity increase depressive symptoms. This result is consistent with an observational cross‐sectional study using data from the US Health Information National Trends Survey, which identified a hyperbolic dose–response relationship between physical activity and general mental health (Y. S. Kim et al. 2012). On the other hand, several previous studies, including that study, demonstrated an optimal range of physical activity of 2.5–7.5 h/week (Y. S. Kim et al. 2012), and that engaging in moderate‐intensity physical activity for 2.5 h/week is sufficient to obtain the benefits of stress buffering and health promotion (Wunsch et al. 2017). In fact, the United States Department of Health and Human Services (HHS) and the World Health Organization (WHO) provide physical activity guidelines in which adults are recommended to perform moderate‐intensity aerobic physical activity for at least 2.5–5 h/week, vigorous‐intensity aerobic physical activity for at least 1.25–2.5 h/week, or an equivalent combination of moderate‐intensity and vigorous‐intensity aerobic physical activities, to achieve significant health benefits (US Department of Health and Human Services, 2018; World Health Organization 2020). In these guidelines, it is also mentioned that performing moderate‐intensity aerobic physical activity for more than 5 h/week, vigorous‐intensity aerobic physical activity for more than 2.5 h/week, or an equivalent combination of moderate‐intensity and vigorous‐intensity activity results in additional health benefits (U.S. Department of Health and Human Services 2018; World Health Organization 2020). Although the guidelines focus on the minimum required duration at each intensity level, and recommend simply increasing physical activity duration for health benefits, these guidelines are not specific to mental health. In the present study, we summed the physical activity duration without distinguishing the intensity (including even walking time) and whether the activities were aerobic or muscle‐strengthening, and concluded that physical activity duration that minimizes depressive symptoms is 25.7 h/week. Although this result is much longer than what is stated in the HHS and WHO guidelines as being required for substantial health benefits, this is not surprising, as it includes physical activities such as walking and housework.

The HHS and WHO guidelines also mention some of the risks and negative health events of too much physical activity. The HHS guidelines state that the risk of adverse events, including musculoskeletal injuries (i.e., injury to bones, muscles, or joints) increases with the total amount of physical activity performed, using the example that an individual who consistently runs 40 miles per week faces a greater risk of injury compared with someone who runs 10 miles per week (U.S. Department of Health and Human Services 2018). The WHO guidelines cites evidence from a commissioned review suggesting an unfavorable association between levels of leisure time physical activity and musculoskeletal injuries (World Health Organization 2020). Although the guidelines refer to the link between the total amount of physical activity or acute physical activity sessions of relatively vigorous‐intensity (e.g., shoveling heavy snow) and negative health events, such as musculoskeletal injuries and cardiovascular events, they do not mention the risk of adverse psychiatric events. The nature of the physical activity is defined based on the total amount and intensity in the guidelines, and it is only concluded that adverse events are generally minimal with moderate‐intensity physical activity, particularly when the increases in physical activity duration, frequency, and intensity are gradual (Physical Activity Guidelines Advisory Committee 2018; World Health Organization 2020). However, they do not explicitly state whether physical activity durations that are too long cause adverse events.

### 4.2 Pathways From Physical Activity to Depressive Symptoms

The additional key finding of this study is that the path analysis indicated that physical activity duration affects depressive symptoms through sleep reactivity, in addition to its direct effect on depressive symptoms. This impact of physical activity duration on depressive symptoms through sleep reactivity was not detected by MLR. To the best of our knowledge, this is the first report proposing that sleep reactivity acts as a mediator in the pathways from physical activity to depressive symptoms. Specifically, we found 2 significant pathways leading from physical activity duration to depressive symptoms via sleep reactivity. In the first pathway, physical activity influences sleep reactivity, which in turn affects depressive symptoms. This pathway, in which sleep reactivity itself mediates the effect of physical activity duration on depressive symptoms, is a notable finding of this study. In the second pathway, physical activity duration influences sleep reactivity, sleep reactivity influences sleep quality, and finally sleep quality affects depressive symptoms. In other words, sleep reactivity mediates the impact of physical activity duration on depressive symptoms by itself and further mediates it through the pathway involving sleep quality. These findings suggest that sleep reactivity is an important factor linking physical activity and depressive symptoms. Sleep reactivity substantially increases the risk of insomnia by modulating the effects of stress‐induced cognitive intrusion (stress‐response) (C. L. Drake et al. 2014), which can exacerbate depressive symptoms. Physical activity may buffer sleep reactivity by changing an individual's body temperature, which in turn activates somnogenic brain areas to initiate sleep (Atkinson and Davenne 2007), thereby improving sleep quality and alleviating depressive symptoms. Interestingly, the pathway leading from physical activity duration to depressive symptoms via sleep quality alone was not significant. This pattern is consistent with findings from our recent study, which suggested that physical activity habits mitigate depressive symptoms, not merely by transiently alleviating anxiety symptoms, but rather by influencing more stable personality characteristics (Kikkawa et al. 2023). More specifically, sleep reactivity is a similar individual factor to trait anxiety in terms of a personality‐like characteristic, which cannot be changed easily. On the other hand, sleep quality has similar properties to state anxiety in that it is a transient condition.

Incidentally, there was a significant direct effect from physical activity duration to depressive symptoms, in addition to indirect effects. This suggests the existence of pathways that are responsible for the impacts of physical activity duration on depressive symptoms, through factors other than sleep reactivity and sleep quality. In fact, we recently reported other factors mediating physical activity duration and depressive symptoms (e.g., trait anxiety, resilience, and neuroticism) in other path models (Kikkawa et al. 2023; K. Nakajima et al. 2023).

### 4.3 Complementary Effects of Physical Activity and Sleep Quality Interventions on Improving Mental Health

In the path analysis of this study, the paths leading to depressive symptoms from both physical activity duration and sleep quality were significant. This suggests that even if an intervention can only target either physical activity or sleep quality, it may have a compensatory effect on patients' depressive symptoms. For example, a previous study provided lines of evidence that moderate‐and‐high level physical activity can alleviate the mental distress experienced by poor sleepers (Zhang et al. 2022). Likewise, the authors claimed that maintaining a healthy sleep status could help compensate psychological well‐being in individuals who are physically inactive.

In the present study, the standardized partial regression coefficient of PSQI (beta = 0.395; *p* < 0.001) was the largest among all independent variables, whereas that of DOP (beta = 0.066; *p* = 0.084) was comparatively small and not significant in MLR. In addition, the standardized coefficient of the total effect of PSQI on PHQ‐9 (*β* = 0.450; *p* < 0.001) was larger than that of the total effect of DOP on PHQ‐9 (*β* = 0.124; *p* = 0.004) in the path analysis. Considering these results, if improving sleep quality is possible, this may be more effective than making changes in physical activity.

### 4.4 Limitations

First, as this study had a cross‐sectional design, the causal associations among the factors could not be determined. For example, because individuals with depression demonstrate a higher cognitive vulnerability to stress, and a propensity to make more negative attributions in response to stressful life events (Haeffel and Grigorenko 2007), there is a possibility that there are causal associations leading from depression to physical inactivity and insomnia, creating vicious cycles. Hence, a prospective follow‐up study should be performed in the future. Second, as the participants of this study were recruited through convenience sampling of adult volunteers from the community, the findings may not reflect the general population, owing to potential sampling bias. The beneficial effects of physical activity may be attenuated in individuals with more severe or longstanding depressive symptoms (Kaseva et al. 2016; Pereira et al. 2013), as well as in those without clinical depression whose depressive symptoms are too mild for the effects of physical activity to be detected (Teychenne et al. 2008). Indeed, the PHQ‐9 score of the participants in this study was 4.0 ± 4.2, suggesting that they had either no or only mild depressive symptoms. These factors should be investigated in future research. Third, the subjective assessments using the self‐administered questionnaires that were applied in this study may diverge from objective assessments. Fourth, although an optimal physical activity duration that minimizes depressive symptoms was identified, an optimal physical activity duration that minimizes sleep reactivity also exists (data not shown). More specifically, not only the relationship between physical activity duration and depressive symptoms but also the association between physical activity duration and sleep reactivity was significantly explained by the quadratic regression, which suggests that both physical inactivity and excessive physical activity will have unfavorable impacts on depressive symptoms and sleep reactivity. However, the optimal physical activity duration for depressive symptoms and sleep reactivity varied slightly, suggesting the need to adjust the optimal duration for both. Finally, the quadratic regression may not fully reflect the actual dose–response association between physical activity duration and depressive symptoms.

## 5 Conclusion

Our study suggests that a deviation from the optimal physical activity duration worsens depressive symptoms by increasing sleep reactivity. One underlying pathway is that physical activity influences sleep reactivity, which in turn affects depressive symptoms. Another pathway is that physical activity influences sleep reactivity, sleep reactivity influences sleep quality, and finally sleep quality affects depressive symptoms.

## Author Contributions

All authors made a significant contribution to the work reported, either in the conception, study design, execution, acquisition of data, analysis and interpretation, or in more than one of these areas, took part in drafting, revising or critically reviewing the article, gave final approval of the version to be published, have agreed on the journal to which the article has been submitted, and agree to be accountable for all aspects of the work.

## Funding

This work was partly supported by a Grant‐in‐Aid for Scientific Research (no. 21K07510 to T.I.) from the Ministry of Education, Culture, Sports, Science and Technology‐Japan, and by the Japan Agency for Medical Research and Development (Grant No. JP23rea522113 to T.I.).

## Ethics Statement

This study was approved by the Institutional Review Committees of Tokyo Medical University (study approval no.: SH3502), in compliance with the Declaration of Helsinki (amended in Fortaleza in 2013).

## Consent

All subjects were informed that participation in this research was voluntary and that the collected information would be anonymized so that individuals could not be identified. Only the subjects who gave their written informed consent to participate in this study were analyzed.

## Conflicts of Interest

A.S. has received personal compensation from Sumitomo Pharma, Nobelpharma, and Eisai. Y.T. has received personal compensation from Otsuka Pharmaceutical, Sumitomo Pharma, Eisai, MSD, and Meiji Seika Pharma. J.M. has received personal compensation from Otsuka Pharmaceutical, Eli Lilly, Astellas, and Meiji Yasuda Mental Health Foundation, and grants from Pfizer. T.I. has received personal compensation from Mochida Pharmaceutical, Takeda Pharmaceutical, Eli Lilly, Janssen Pharmaceutical, MSD, Taisho Toyama Pharmaceutical, Yoshitomiyakuhin, and Daiichi Sankyo; grants from Shionogi, Astellas, Tsumura, and Eisai; and grants and personal compensation from Otsuka Pharmaceutical, Dainippon Sumitomo Pharma, Mitsubishi Tanabe Pharma, Kyowa Pharmaceutical Industry, Pfizer, Novartis Pharma, and Meiji Seika Pharma; and is a member of the advisory boards of Pfizer, Novartis Pharma, and Mitsubishi Tanabe Pharma. The other authors declare that they have no actual or potential conflicts of interest associated with this study.
